# P85 An audit of the appropriateness of systemic antifungal prescribing for non-haematology/oncology indications

**DOI:** 10.1093/jacamr/dlag102.091

**Published:** 2026-06-26

**Authors:** Amy Fleming, Teig Parsons, Rachel Tan

**Affiliations:** Pharmacy Department, Oxford University Hospitals NHS Foundation Trust, Oxford, UK; Antimicrobial Stewardship Team, Oxford University Hospitals NHS Foundation Trust, Oxford, UK; Antimicrobial Stewardship Team, Oxford University Hospitals NHS Foundation Trust, Oxford, UK

## Abstract

**Background:**

Fungal infections are an increasing global health concern, particularly in the immunocompromised group. There are currently only four classes of antifungals that are available, with a few others under development.^1^ Optimizing antifungal use is essential to maintain drug efficacy and prevent antifungal resistance. Antifungals are also associated with significant toxicity and drug interactions therefore it is important to monitor and review their use.

**Objectives:**

To assess the appropriateness of systemic antifungal prescribing with respect to indication, choice, dose and duration according to local antimicrobial guidelines. Audit standards included: indication is as per infection specialist advice or in line with treatment guidelines: ≥90%; dose is appropriate for patient's renal/hepatic function: ≥90%; dose is appropriate for patient's weight (if weight-based dosing is required): ≥90%; and duration of antifungal therapy is appropriate as per guidelines or clinical documentation: ≥90%.

**Methods:**

A retrospective audit was conducted of systemic antifungal prescribing for adult (≥18 years) inpatients across non-ICU, non-haematology/oncology specialties within a large teaching hospital. Data was collected from the electronic prescribing system (EPR, Cerner Millenium) looking backwards from 01/10/2025 until 40 eligible patients were found. Antifungals included were fluconazole, voriconazole, itraconazole, isavuconazole, posaconazole, caspofungin, amphotericin B and flucytosine for the treatment of superficial and invasive fungal infections. Prescriptions were assessed for appropriateness of indication, loading and maintenance doses, duration, therapeutic drug monitoring (TDM), adverse drug reactions (ADRs), and clinically significant drug–drug interactions.

**Results:**

The majority of antifungal prescriptions originated from general medicine (31.0%). Fluconazole was most frequently prescribed (64.3%), primarily for oropharyngeal and/or oesophageal candidiasis (55.5%). Choice of antifungal is in line with guidelines or infection specialist advice, except for dermatophyte infections (4.8%). Pulmonary aspergillosis comprised the majority (25.0%) of invasive fungal infections. Dosing was appropriate for renal function (95.2%) and weight (97.6%). Loading courses were prescribed accurately for most patients. There were two patients who were unnecessarily loaded on fluconazole for oropharyngeal candidiasis, a patient who was not prescribed a loading dose and a patient who received a partial loading course. Duration was mostly guideline-compliant or based on infection specialist advice (83.3%), although a few fluconazole courses for superficial fungal infections exceeded recommended course length. TDM was undertaken for all patients where indicated; however, sampling timing errors were common, with samples frequently taken post-dose rather than as pre-dose trough levels. Prescriptions involved significant (as defined by Stockley’s Drug Interaction database) azole–statin interactions and citalopram. ADRs reported included visual hallucinations and photosensitivity with voriconazole, and hepatotoxicity with fluconazole.

**Conclusions:**

Overall, systemic antifungal prescribing was appropriate and aligned with local guidelines. Two key improvement areas were identified: (1) timing of TDM sampling and (2) management of azole–drug interactions. Education sessions on pre-dose trough sampling and EPR prompts to support correct timing are recommended. Prescribers should also be reminded to review potential interactions, particularly between azoles and statins, to reduce the risk of myopathy and rhabdomyolysis.
